# Safety Properties of *Escherichia coli* O157:H7 Specific Bacteriophages: Recent Advances for Food Safety

**DOI:** 10.3390/foods12213989

**Published:** 2023-10-31

**Authors:** Bukola Opeyemi Oluwarinde, Daniel Jesuwenu Ajose, Tesleem Olatunde Abolarinwa, Peter Kotsoana Montso, Ilse Du Preez, Henry Akum Njom, Collins Njie Ateba

**Affiliations:** 1 Food Security and Safety Focus Area, Faculty of Natural and Agricultural Sciences, North-West University, Mahikeng 2375, South Africa; bukkyopeyemi@yahoo.com (B.O.O.); ajosedj@yahoo.com (D.J.A.); abolarinwatesleem@yahoo.com (T.O.A.); montsokp@gmail.com (P.K.M.); 2 Antimicrobial Resistance and Phage Bio-Control Research Group (AREPHABREG), Department of Microbiology, North-West University, Mahikeng 2735, South Africa; 3 Centre for Human Metabolomics, North-West University, Potchefstroom 2531, South Africa; ilse.dupreez@nwu.ac.za; 4 Agricultural Research Council, Private Bag X1251, Potchefstroom 2531, South Africa; njomh@arc.ac.za

**Keywords:** food borne infection, antimicrobial resistance, *Escherichia coli* O157:H7, phage therapy, whole genome sequencing, one health approach

## Abstract

Shiga-toxin-producing *Escherichia coli* (STEC) is typically detected on food products mainly due to cross-contamination with faecal matter. The serotype O157:H7 has been of major public health concern due to the severity of illness caused, prevalence, and management. In the food chain, the main methods of controlling contamination by foodborne pathogens often involve the application of antimicrobial agents, which are now becoming less efficient. There is a growing need for the development of new approaches to combat these pathogens, especially those that harbour antimicrobial resistant and virulent determinants. Strategies to also limit their presence on food contact surfaces and food matrices are needed to prevent their transmission. Recent studies have revealed that bacteriophages are useful non-antibiotic options for biocontrol of *E. coli* O157:H7 in both animals and humans. Phage biocontrol can significantly reduce *E. coli* O157:H7, thereby improving food safety. However, before being certified as potential biocontrol agents, the safety of the phage candidates must be resolved to satisfy regulatory standards, particularly regarding phage resistance, antigenic properties, and toxigenic properties. In this review, we provide a general description of the main virulence elements of *E. coli* O157:H7 and present detailed reports that support the proposals that phages infecting *E. coli* O157:H7 are potential biocontrol agents. This paper also outlines the mechanism of *E. coli* O157:H7 resistance to phages and the safety concerns associated with the use of phages as a biocontrol.

## 1. Introduction

*Escherichia coli* (*E. coli*) is one of the most thoroughly investigated and characterized living organisms and is widely employed as a research model [1]. Despite the fact that these organisms are known to exist as normal flora in the gastrointestinal tract of humans and other warm-blooded animals, some strains are considered to be pathogenic [2]. In general, *E. coli* strains linked to human illnesses can be divided into two categories, namely intestinal *E. coli*, also known as diarrheagenic *E. coli*, and extra-intestinal *E. coli* [3]. Based on their distinctive virulence traits, pathogenic mechanisms, and clinical symptoms, intestinal *E. coli* strains are further separated into six pathotypes [4] comprising enterotoxigenic *E. coli* (ETEC), enteropathogenic *E. coli* (EPEC), enteroaggregative *E. coli* (EAEC), enteroinvasive *E. coli* (EIEC), diffusely adherence *E. coli* (DAEC), and enterohaemorrhagic *E. coli* (EHEC) [3,4]. According to Kaper et al. [4], uropathogenic *E. coli*, meningitis-associated *E. coli*, and necrotoxigenic *E. coli* are the three types of strains associated with extra-intestinal infections.

There are other significant EHEC serotypes, such as O104:H21 and O111:H8, that cause diseases in humans; however, strains belonging to the serotype O157:H7 are major foodborne pathogens that pose serious threats to public health globally [5,6]. These pathogens cause a number of complications ranging from mild diarrhoea to more potentially fatal haemolytic uraemic syndrome (HUS), haemorrhagic colitis (HC), and thrombotic thrombocytopenic purpura (TTP), accounting for a large proportion of kidney failure [7]. To date, South Africa has not experienced any documented food-borne outbreaks linked with *E. coli* O157. However, previous surveillance studies have established a significant genetic correlation between *E. coli* O157 strains obtained from various sources such as water, cattle, their associated meat products, and individuals suffering from diarrheal illnesses [8,9].

Furthermore, recurring and long-lasting outbreaks of foodborne illnesses which are caused by alterations in the pattern of pathogen population have a significant impact on human health and safety [10,11]. Reports from countries, such as the USA, that have more advanced public health policies reveal that *Listeria monocytogenes* (28%) and *E. coli* O157:H7 (3%) are responsible for a significant proportion (30%) of food-related deaths [12]. The transmission of *E. coli* O157:H7 is associated with its potential to survive in a wide range of environmental conditions and contaminate the food chain along the preharvest and postharvest production pipelines through multiple routes [13,14]. Also, it is not surprising that, on the WHO global priority pathogens (GPP) list, antimicrobial resistant *E. coli* O157:H7 is categorized as a critical pathogen requiring constant monitoring and surveillance [15]. Although other non-O157 serogroups, such as O26, O111, O103, O121 O45, and O145, have also been increasingly associated with foodborne illness in humans, serotype O157:H7 is predominantly known to be the causative agent of STEC infections worldwide [16]. In addition, foodborne outbreaks are increasingly associated with fresh produce, like organic fruits and vegetables [17], which are partially cooked or mainly consumed raw with no inactivation step like heating. The implementation of comprehensive strategies to prevent the transmission of *E. coli* O157:H7 throughout food production systems, without altering the colour, taste, texture, or nutritional characteristics of food products, at both pre and post-harvest facilities, is imperative.

Treatment of *E. coli* O157:H7 infections in humans and animals with antibiotics is a popular and major option [18]. However, the use of antimicrobial agents in the treatment of *E. coli* O157:H7 infections is controversial [19]. Certain antibiotics can stimulate *E. coli* O157:H7 to release toxins that can potentially worsen the clinical outcome of infected patients, e.g., through an elevated risk of HUS, and also the use of antimicrobials may contribute to the development of antibiotic-resistant strains, thereby broadening public health implications. The growing prevalence of antimicrobial resistance (AMR) contributes to the severity of *E. coli* O157:H7 infections.

Furthermore, *E. coli* O157:H7 populations with multiple drug resistance (MDR), extensive drug resistance (XDR), and pan drug resistance (PDR) are rapidly emerging [20,21]. The primary cause of this resistance is the improper and extensive use of antimicrobial drugs in both humans and animals [22]. The rise in antibiotic resistance in *E. coli* O157:H7 is a global health concern, resulting in high mortality and morbidity, prolonged infection durations in vulnerable persons and animals, and significant economic losses [23].

In addition, live animals, particularly cattle, harbour foodborne pathogens, such as *E. coli* strains that can be transmitted to other animals, that can enter the food chain and thus be transmitted to humans. There is a need to develop alternative but effective methods for reducing the quantity of pathogens in live animals [24]. Moreover, strategies to reduce faecal shedding of these organisms by animals is highly recommended [25,26]. To date, various decontamination strategies, such as the use of chemical sanitizers (sodium dichloroisocyanurate, quaternary ammonium compounds, chlorine, peracetic acid, and lactic acid), heat treatment (pasteurization), washing (water), and chilling, are commonly used during food processing to reduce the risk of pathogens entering the food chain [27,28]. These strategies have limited, but sometimes direct, microbial benefits on the food product [29].

The rise in antimicrobial resistance, as well as the difficulties associated with antibiotic use for *E. coli* O157:H7 infections, highlights the critical need for alternative novel approaches. Such approach should not only target *E. coli* O157:H7 but also the control the development and spread of antibiotic resistance. Bacteriophage-based interventions are being regarded as promising biocontrol agents in the food production systems despite the perceived shortcomings associated with phage-based control measures.

Bacteriophages (phages) are bacteria viruses that infect and kill their host (bacteria). They are the most abundant entities in the biosphere, with approximately 10^31^ phage particles, which is ten times more than the number of bacteria species on earth [30]. Based on their life cycle, phages are classified into two major groups, namely, lysogenic and lytic phages. The current review is focused on lytic phages due to their antibacterial properties. The process of phage infection involves phage attachment to the bacteria cell. Upon entry into the host cell (bacteria), lytic phages hijack the genetic material of the host bacteria in order to generate new virions by taking over the bacterial cell replication mechanism [31]. Subsequently, phages employ late proteins, such as lysozyme (lysin and/or endolysin), to lyse (kill) the host (bacteria) cell and thus release the mature virions. Owing to their ability to lyse the bacteria, lytic phages has attracted interest in the pharmaceutical industry. The use of lytic bacteriophages is one innovative strategy that is gradually being embraced as a green technology to combat antimicrobial resistance and the risk of illness in humans [32,33]. More importantly, the proliferation of bacteriophage-insensitive mutants (BIMs) and save our soul (S.O.S) in response to Shiga toxin production are some of the safety concerns associated with using phages as bio-control agents [34,35]. Hence, it is crucial to establish the efficacy and biosafety of these promising biocontrol and therapeutic agents. In this review, we describe the advantages of phages as an alternative antibacterial agent and how genomics has enhanced investigations aimed at assessing the safety of phage therapy, but with a direct focus on *E. coli* O157:H7-specific bacteriophages.

## 2. Evolution, Virulence and Pathogenicity of *E. coli* O157:H7

Non-toxigenic and less virulent *E. coli* O55:H7 strains are known to be ancestral cells of *E. coli* O157:H7 [36,37] that originated through a series of transitional phases [38]. According to the concept model, the locus of enterocyte effacement (LEE) was present in both the O55:H7 and O157:H7 strains, and it is capable of inducing diarrhoea through an attachment–effacement process [39,40,41]. Four sequential events that led to the emergence of *E. coli* O157:H7 include (i) acquisition of an *stx* operon, which is encoded in the genome of lambdoid prophages, (ii) acquisition of the *rfb* region encoding the O157 antigen, and (iii) loss of β-glucuronidase (GUD) activity [42,43]. Notably, the three main virulence factors of *E. coli* O157:H7 include (i) Shiga toxin operons, (ii) locus of enterocyte effacement, and (iii) byproducts of the F-like plasmid pO157 [44,45].

Based on the degree of its virulence, the Shiga toxin operon is divided into two major groups, namely, *stx*_1_ and *stx*_2_ genes [46]. Interesting, the Stx1 has three subtypes (Stx1a, Stx1c, and Stx1d), while Stx2 has seven subtypes (Stx2a, Stx2b, Stx2c, Stx2d, Stx2e, Stx2f, and Stx2g). It is widely acknowledged that diverse stx subtypes possess the ability to bind to multiple receptors, exhibiting varying affinities for each receptor. The primary target for Stx2a is the Gb3 receptor, whereas Stx1a can bind to both Gb3 and globotetraosylceramide (Gb4). The Stx2e subtype exhibits a broader spectrum of receptor binding capabilities, including interactions with globopentaosylceramide, pentahexosylceramides featuring Gb4-elongated core structures, and Gb4 with a preference for Gb5 [47]. Several other genes, apart from *stx*, have been associated with the pathogenicity and virulence of O157:H7 [48]. These genes differentiate *E. coli* O157:H7 from non-pathogenic strains of *E. coli* and are presented in Table 1.

### 2.1. Transmission of E. coli O157:H7 to Humans and Animals

**Figure 1:**
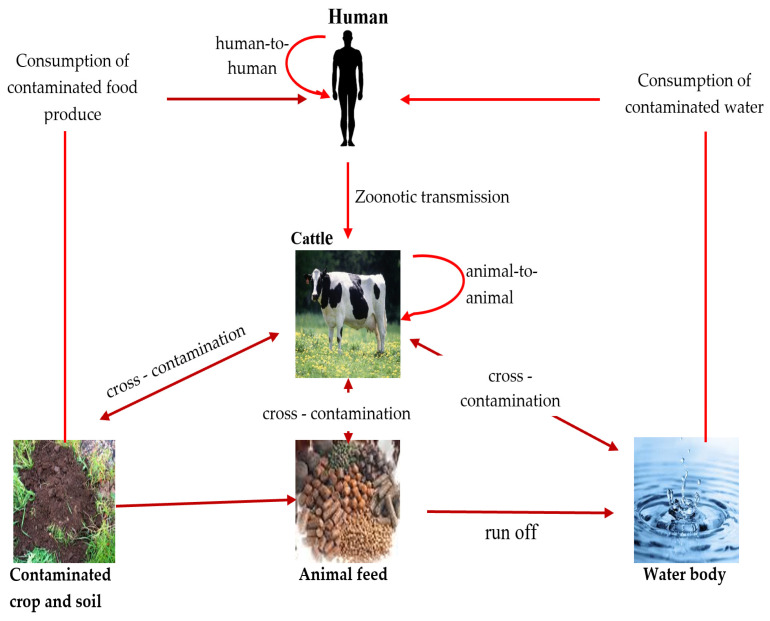
Transmission route of pathogenic *E. coli* among animal, human, and environment (source: this study—diagram prepared by authors).

*E. coli* serotype O157 can be transmitted through different routes, including direct contact with animal droppings, ingestion of contaminated food and water, and transmission from one person/animal to another [5,49] (Figure 1).

#### 2.1.1. Contaminated Food as a Transmission Vector

Contaminated food plays a crucial role in the transmission of *E. coli* O157:H7 to humans [50]. Food products derived from animals (beef, lamb, chicken, pork, and ground beef) are considered to be the most common source of the pathogen and the transmission of the diseases amongst humans worldwide [51]. Studies have reported that *E. coli* O157:H7 illnesses have also been linked to the ingestion of other food products originating from cattle, such as unpasteurized milk and other dairy products [9,52]. Furthermore, the transmission of this pathogen from the skin, intestines, and excrement of diseased animals frequently leads to the contamination of the environment, including water sources [5]. As a result, consumption of water contaminated by *E. coli* may lead to illness in humans.

Fresh vegetables such as alfalfa, radish sprouts, lettuce, and spinach, in addition to unpasteurized fruit juices and apple cider [53,54,55], have been connected to outbreaks of *E. coli* O157-related disease in humans. It is thought that these fresh vegetables become contaminated when grown in soil that was exposed to infected animal dung or polluted water [9], while fruit juices and apple ciders become contaminated due to improper manufacturing processes [55]. In general, animal reservoirs are responsible for the greatest amount of *E. coli* O157:H7 transmission to humans, and food-related transmission is common, making it necessary to manage the pathogens in animal-derived foods, especially beef. As a result, *E. coli* O157:H7 poses a major risk to public health by endangering both human health and food safety.

#### 2.1.2. Contaminated Water as a Transmission Vector

The transmission of *E. coli* O157:H7 through water has been documented in various settings, including both drinking water that has been contaminated [56] and recreational water bodies [57,58]. Furthermore, studies conducted in Nigeria and South Africa demonstrated that water used for irrigation had notable influence on the dissemination of *E. coli* O157:H7 through the contamination of food products [59,60].

Untreated sewage released from hospitals, farms, and residential areas containing *E. coli* O157:H7 into nearby water bodies also increases the risk of human infections significantly [61]. Based on the evidence, *E. coli* O157:H7 easily survives in water and may persist for several weeks and even longer [58]. Strong rainstorms may cause sediments to be re-suspended, which could lead to an abrupt rise in *E. coli* O157:H7 concentrations in the water [62]. Xie et al. [58] reported the detection of *E. coli* O157 in urban recreational water and provided evidence of the correlation between contact with these water bodies through activities such as swimming, boating, bathing, and sailing and the development of infections. In addition, an outbreak of *E. coli* O157 infections occurred among a group of seven children in the United Kingdom who came in contact with recreational water at a coastal beach [63]. The pathogens originated from water in a creek that was contaminated with faeces from cows grazing upstream, and the transmission was facilitated by rainfall runoff [63]. This finding highlights the potential vulnerability of humans when in contact with recreational beach environments.

#### 2.1.3. Person-To-Person Transmission

The transmission of *E. coli* O157:H7 infection most often occurs through the faecal–oral route, primarily in settings where infected individuals come in close contact with others, such as in households, daycare centres, and healthcare facilities [64,65]. Children are more vulnerable than adults due to their immunological immaturity and limited understanding of appropriate hygiene practices [66]. In numerous instances, there has been a correlation between the consumption of shared contaminated food or water and the occurrence of an outbreak, thereby emphasizing the importance of implementing strict control measures in healthcare facilities and hygiene practice amongst people living in shared apartments.

### 2.2. Epidemiology of E. coli O157:H7

According to Mesele and Abunna [67], *E. coli* O157:H7 is responsible for approximately 73,000 illnesses, 2000 hospitalizations, and 50–60 deaths annually in the United States. Reports that evaluated *E. coli* O157 outbreaks that occurred in the USA between 1982 and 2002 and were reported to the Centers for Disease Control and Prevention (CDC) showed 350 outbreaks, totalling 8598 cases and 1493 (17%) hospitalizations. A total of 354 (4%) presented with HUS and 40 (0.5%) died [68]. This highlights the significant public health potential of *E. coli* and also provides an overview of its epidemiology in the region.

The varying aetiology of *E. coli* O157:H7 infections explains why the pathogen has also been isolated in humans, animals, food products, and the environment, especially in the African region [69], where public health policies are not adhered to strictly. Examples of documented cases in Africa are as follows: human infection that was reported in Johannesburg, South Africa in 1990 [70]; the detection of the pathogen in individuals with haemorrhagic colitis that resulted to fatalities in Bangui, Central African Republic [71]; and the isolation of the pathogen in East African countries (Tanzania, Kenya, and Ethiopia) [72]. The prevalence rates of STEC O157:H7 reported in Africa were 7% in Morogoro, Tanzania, amongst patients suffering from diarrhoea [72], 2.3% isolated from raw cattle milk in Kwara, Nigeria [73], 1.9% amongst children with diarrhoea in Mozambique [74], and 8% in HIV infected individuals suffering from dysentery in Zimbabwe [75].

**Table 1:** Key virulence factors associated with *E. coli* O157:H7 and their function in pathogenesis.

Virulence Factor	Gene	Role in Pathogenesis	References
Shiga toxin	*stx*1 (a, c, and d) and *stx*2 (a to g)	Stx toxin is composed of 2 subunits, A and B. The A subunit is an RNA-glycosidase that interacts with the 60S rRNA, inhibiting protein synthesis, causing cell death.	[76]
Locus of Enterocyte Effacement (LEE) pathogenicity island (PAI)	*lee*	LEE-PAI encodes several genes like *eha*A, *eha*B, and *eha*J, which are involved in the adhesion mechanism called the attaching-and-effacing (A/E) lesion, such as type III secretion system (TTSS), an outer membrane adhesion (intimin), its translocated intimin receptor (TIR), and secreted proteins with signal transduction.	[77]
Intimin	*eae*	The eae gene is carried by the LEE-PAI and encodes an outer membrane adhesion protein called Intimin. This protein interaction with the bacterial translocated intimin receptor (Tir) leads to the attachment of the STEC cell to the host intestinal mucosa, resulting in the A/E lesions	[76,78]
EHEC hemolysin (EHEC-*hly*)	*ehx*A or EHEC-*hly*A	EHEC-*hlyA* is part of an operon composed of four genes (EHEC-*hlyC*, EHEC-*hlyA*, EHEC-*hlyB*, and EHEC-*hlyD*) encoded on the pO157 plasmid. EHEC-*hlyA* codes the structural protein of EHEC-Hly. The proteins coded by EHEC-*hlyB* and EHEC-*hlyD* are responsible for the transport of EHEC-Hly out of the bacterial cell, and, in its turn, EHEC-*hlyC* product is responsible for EHEC-Hly post-translational activation. EHEC-Hly is responsible for forming pores into the cell membrane and is found as free or in association with outer membrane vesicles (OMV). Both forms target the human intestinal epithelial and microvascular endothelial cells, but in different manners. When in the free form, EHEC-Hly is responsible for lysing the cells, but when in association with OMV, induces cell apoptosis.	[79]
Adhesins	*eibG, efa-1/lifA/toxB*	Immunoglobulin-binding (Eib) G is encoded by the gene *eibG*, and it can bind to human IgD and IgA. It can also participate in bacterial adhesion to host epithelial cells. Toxin Efa-1 is like LifA and ToxB, and they are thought to be associated with cell adherence, lymphostatin activity, or induction of secretion of type III effectors in the STEC strain.	[78]
Serine protease autotransporters	*esp*P	Encoded on the pO157 plasmid, it is important for biofilm formation and for adherence to T84 intestinal epithelial cells.	[78]
Long Polar Fimbriae and *E. coli* YcbQ laminin-binding fimbriae (ELF)	lfp and elf	Able to attach the extracellular matrix protein laminin, which contributes to colonization of the GI tract	[76]

Adapted with permission from: Pinto et al. [80], 2023, Taylor & Francis Ltd., http://www.tandfonline.com, 1 September 2023.

### 2.3. Treatment for E. coli O157:H7 Infections and Antimicrobial Resistance

Antibiotics are frequently used in animal production to prevent infection and as a growth promoter. This practice has made it difficult to treat infections caused by *E. coli* O157:H7, as the pathogens have become resistant to antibiotics. Given the virulence potential and pathogenicity of *E. coli* O157:H7, it is unfortunate that there is no specific treatment for *E. coli* O157:H7 infection [67]. In humans, antibiotics are typically not recommended because they can increase the risk of complications, such as HUS, a serious kidney disease. Although the spread of antimicrobial resistance in *E. coli* O157:H7 and other pathogens is being monitored and mitigated by surveillance [81], an alternative treatment method is necessary as a strategy to combat *E. coli* O157:H7 and infections caused by it, and also to serve as an additive to feed stock for the prevention of sicknesses. Bacteriophage therapy may be an effective alternative to antibiotic therapy for antibiotic-resistant *E. coli* O157:H7, both in humans and in animal production.

## 3. Bacteriophages

Bacteriophages are viruses that exclusively infect bacteria. Although there were arguments regarding the discovery of phages, it has been acknowledged that the first scientists to discover the phages were William Twort and Felix d’Herelle [82,83]. Twort, an English medical bacteriologist, postulated that viruses were the causative agents of this phenomenon after observing a similar process in Micrococcus [84,85,86]. According to d’Herelle’s findings, the presence of an imperceptible microorganism was detected in stool samples obtained from dysentery patients, which were devoid of bacteria due to filtration [87]. The occurrence of clear zones observed in bacterial lawns can be attributed to the presence of “invisible germs”. D’Herelle provided the initial elucidation regarding the mechanism by which these viruses proliferate by exploiting bacteria, resulting in the death and subsequent disintegration of the bacteria. After their discovery, d’Herelle used phage preparations to treat bacterial dysentery [88]. Owing to its success in treating bacterial infection, the use phage therapy escalated in countries such as Tbilisi, Georgia, and Poland to treat conditions such as typhoid, fever, dysentery, surgical wound infections, peritonitis urinary tract infection, and septicaemia [89]. However, the use of phage therapy was halted by the discovery of the first antibiotic, penicillin, in 1940. In addition, lack of knowledge of phage therapy decreased the use of phage therapy. Despite this, Soviet countries continued to use phages to treat bacterial infections in humans.

Bacteriophages are ubiquitously found in the vicinity of their host bacteria. The phage replication cycle encompasses the processes of adsorption, penetration, nucleic acid replication, and virion assembly. Consequently, the cellular membrane undergoes lysis, leading to the release of mature virions. While it is true that certain phages can infect multiple bacterial species or strains, most phages exhibit specificity towards a particular bacterial species or strain. The determination of specificity is regulated by the existence of receptors located on the outer surface of bacterial cells, such as lipopolysaccharide (LPS), flagella, and/or other surface proteins [90]. Bacteriophages can exhibit either a lytic or lysogenic life cycle, depending on the specific life cycle they adopt after infecting a host cell. Lytic phages undergo a lytic cycle, which starts when the bacteriophage binds to specific receptors on the surface of the bacterial cell. The phage then injects its DNA into the host cell and assumes control of the cellular machinery. While using the host cell’s transcription and translation mechanisms, phage-specific proteins are synthesized, and the phage’s DNA is replicated. The components then assemble into new phage particles, and they accumulate and exert pressure on the cell’s internal environment, causing the bacterial cell to lyse or rupture. In the lytic lifecycle of a bacteriophage, the lysis process is a crucial phase. It enables the newly produced phage particles to disseminate and infect other susceptible bacterial cells, ultimately resulting in the phage population increasing. Temperate phages, on the other hand, undergo the lysogenic cycle, wherein their genetic material integrates and coexists with the host genome in a stable manner, forming a prophage. This prophage replicates along with the host cell during replication. According to Pinto et al. [80], temperate phages have the ability to transition into the lytic cycle when they encounter cellular stress.

### 3.1. General Properties of Bacteriophages

The attributes of interest possessed by phages as an antibacterial agent have generated considerable interest in using them as a biocontrol agent [91]. The aforementioned attributes encompass the following: firstly, phages demonstrate selective toxicity by exclusively targeting their host, such as the food-borne pathogen *E. coli* O157:H7, while leaving the indigenous microflora of the food substance unharmed; secondly, their low toxicity to humans stems from their predominant composition of nucleic acids and proteins; and thirdly, phages naturally exist in the environment, rendering them relatively straightforward and cost-effective to manufacture. Phages have been identified in chicken meat [92], seafood [93], fermented products [94], and different human body parts, which provides further evidence supporting the safety of phages in human applications [95]. Despite the advantageous characteristics exhibited by phages, they also possess unfavourable attributes that present obstacles in their application for biocontrol purposes [96]. Brovko et al. [96] and Bandara et al. [97] have identified several key traits associated with these phages. These include a restricted host range, the ability for virulence components to be transferred among bacterial strains, and the emergence of phage-resistant bacterial species.

Furthermore, bacteriophages have been used as a means to eliminate foodborne illnesses for several years [98]. According to O’Sullivan et al. [99], lytic phages have practical applications in ensuring food safety. Phages are commonly regarded as natural entities [100,101], in contrast to alternative food preservatives that have been associated with significant health issues such as cancer, asthma, and other ailments [102]. Jones et al. [103] posed that phages administered to food products for the purpose of eradicating a particular infection does not exhibit significant impact on non-target bacteria, like the commensal microorganisms present in the human body. There is currently no evidence to suggest that they pose any harm to mammalian cells, as they exhibit a high degree of specificity towards a particular species and so they are extensively utilized, even in food products. According to Ramos-Vivas et al. [104], they have been recognized for their efficacy in combating biofilm structures while also maintaining the sensory properties of food.

### 3.2. Characteristics of E. coli O157:H7 Phages

**Figure 2:**
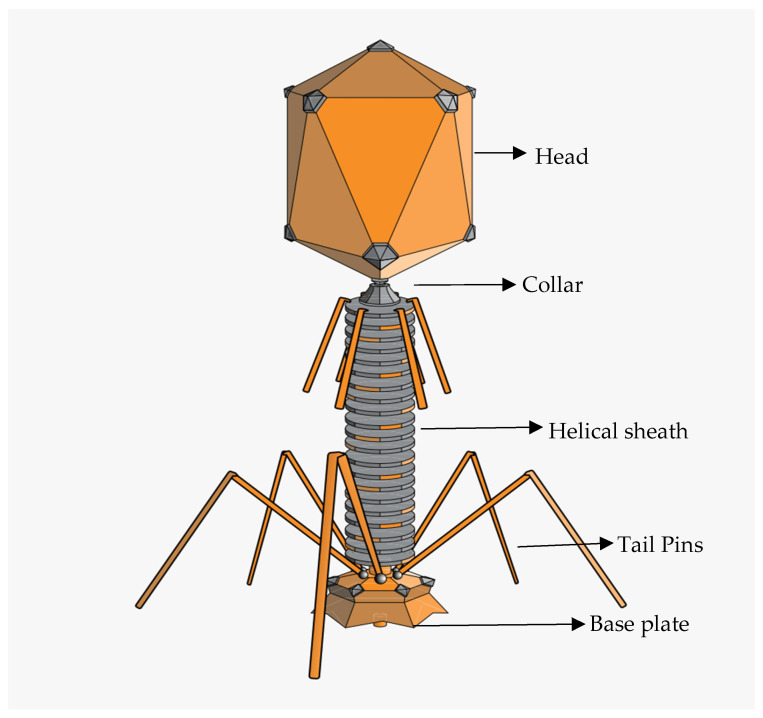
Diagrammatic representation of tailed phage (https://en.m.wikipedia.org/wiki/Bacteriophage, accessed on 1 September 2023).

Being a pathogenic strain, *E. coli* phages can be targeted by various bacteriophages (phages) with different characteristics in terms of their biological, structural, and physicochemical characteristics. A typical phage particle structure consists of a three-dimensional shape made up of an icosahedral protein capsid with or without a tail and a filament [105]. Phage particles can range in size from 24 to 200 nm [106,107], with the largest being the T4 phage, which infects *E. coli* and is 200 nm long and 80–100 nm wide [108]. The physical properties of phages specific to *E. coli* O157 can vary depending on their family. They do, however, often exhibit the conventional phage structure (Figure 2), with an icosahedral head (capsid) carrying the phage’s genetic material, either DNA or RNA. The tail, which aids phage adherence to the bacterial surface and the transfer of genetic material into the host cell, varies throughout phage families and can be lengthy, flexible, or short. Certain *E. coli* O157 bacteriophages have unique tail fibres or tailspikes that aid in targeted binding to receptors on the surface of *E. coli* O157 cells.

The International Committee on Taxonomy of Viruses (ICTV) and the Bacterial and Archaeal Subcommittee (BAVS), primarily dedicated to the study of phages, play pivotal roles in classifying viruses and assigning taxonomic names to virus groups [109]. This classification process relies on evaluating several key characteristics of viruses, including the nature of the viral genome (single-stranded or double-stranded DNA or RNA), the composition of the capsid (the protein shell) and the presence or absence of an envelope, the range of hosts a virus can infect, its pathogenicity, and its genetic sequence similarities. However, in 2022, changes were made to the taxonomy of bacterial viruses, which involved the abolishment of the morphology-based families *Podoviridae*, *Siphoviridae*, and *Myoviridae*, as well as the order *Caudovirales*, therefore establishing a binomial system of nomenclature for species [110]. All tailed bacterial and archaeal viruses with icosahedral capsids and a double-stranded DNA genome are now grouped under the class “*Caudoviricetes*”. With 14 new families assigned to 4 orders, the process of grouping is still ongoing; hence, most taxa have remained “unclassified” at this level.

The most common lytic phages, which includes the *E. coli* O157 phage, associated with human pathogens and the gut microbiota belongs to the class *Caudoviricetes* [111], commonly known as “tailed phages”, which contain double-stranded DNA genomes. They can belong to several families and orders, which can be confirmed via genetic similarities using online tools for phylogenetic similarities [112].

### 3.3. The Application of Bacteriophages in Food Production as Biocontrol Agents

In recent times, bacteriophages have demonstrated a diverse array of applications in enhancing the safety of various food products (Table 2). In the early stages of phage discovery, a pair of researchers affiliated with Michigan Agricultural College identified a substance with inhibitory properties in the liquid derived from decomposing cabbage. This substance effectively impeded the spoilage of vegetables caused by the organism *Xanthomonas campestris pv. Campestris* [113]. From then on, bacteriophages have been systematically evaluated for their efficacy in combating various bacterial species responsible for plant spoilage, with experiments conducted in controlled laboratory conditions as well as real-world environments [114]. In a study conducted by Das et al. [115], it was demonstrated that grapevines artificially infected with *Xylella fastidiosa*, the causal agent of Pierce’s disease, were treated with high-concentration combination of four lytic phages. This treatment was administered three weeks after the initial inoculation of the pathogen, it was observed that the phage cocktail was able to significantly lower the level of *Xylella fastidiosa*. Bacterial pathogens that pose a potential threat to agricultural animals can lead to adverse alterations in the quality of food products like meat and unpasteurized milk, thereby resulting in production losses. Therefore, the investigation into the efficacy of phages in the treatment of animal infections and in the enhancement of animal health and growth has become popular.

In addition to the analysis of food samples, various bacteriophages have also been evaluated in environmental samples associated with food processing facilities. Biofilms play a crucial role in the context of the food industry. In their study, Dalmasso et al. [116] conducted an assessment of the potential anti-biofilm properties exhibited by three recently discovered bacteriophages. The efficacy of the three bacteriophages in combating biofilms were assessed individually as well as in combination. The use of a three-phage cocktail has been identified as a highly effective strategy for the management of biofilms.

**Table 2:** An overview of *E. coli* O157:H7-specific phages, their application on food products and food contact surfaces, their mode of administration, and the obtained results.

Phages	Product Tested	Methodology	Results	References
BEC8 cocktail (38, 39, 41, CEV2, AR1, 42, ECA1, and ECB7)	Spinach leaves and romaine lettuce.	Bacteria were spot inoculated onto the leaves and allowed to dry for 1 h in a biosafety cabinet. BEC8, Transcinnamaldehyde, or TSB were applied on the top of the leaf inoculated previously. Positive controls were prepared by mixing the bacterial inoculum with BEC8 or TC without drying.	Both BEC8 and Transcinnamaldehyde were able to reduce the cell counts at different MOIs and temperatures. The loss of viability of high inoculum levels of *E. coli* O157:H7 was less than 1 log CFU at all conditions with the exception of 3 log CFU after 24 h at room temperature and 37 °C for both leafy greens. The combination of both agents resulted in an increase in the antimicrobial effect.	[117]
Phage OSY- SP	Green bell pepper and spinach	Both matrices were spot inoculated with *E. coli*. The phage was applied by rinsing the food products with phage lysate (for optimization) and phages suspended in PBS before storing. A rinse of 2 min was used for spinach leaves; however, due to the migration of cells in the pepper, a 5 min rinse was then selected for this kind of matrix.	Cell reduction was observed during refrigerated storage conditions. *E. coli* O157:H7 was reduced by 2.4–3.0 log CFU/g on cut green pepper (5 min rinse) and 3.4–3.5 log CFU/g on spinach leaves (2 min rinse) during 72 h storage. The rinse treatment with phages was successful in both fresh produces tested.	[118]
A cocktail composed by the phages e11/2 and e4/1c	Cattle hide	The phage cocktail was administered using a hand-held spray bottle. As negative control, no wash treatment was performed.	The phage cocktail was more efficient when applied to the cattle hide and left for 1 h. There was a 1.5 log_10_ CFU/cm^2^ reduction in *E. coli* O157:H7 colonies compared to the colonies recovered on samples treated with water only.	[119]
BEC8 cocktail	Sterilized hard surfaces (stainless steel chips, ceramic tile chips, high density polyethylene chips—HDPEC).	Bacteria were spotted on the chip and dried in a biosafety cabinet. BEC8 or TSB were applied on the chip surface previously inoculated. MOIs used were 1, 10, and 100. Positive controls were prepared by mixing the bacterial inoculum with BEC8 or TC without drying.	Phage cocktail could inactivate the bacterial mixture with higher performance rating from low to high MOIs, low to high temperatures, and shorter to longer periods of exposure. With a reduction of at least one log CFU in the number of the *E. coli* O157:H7 cells, and at approximately 10^4^ CFU, no survival was detected.	[120]
Wild-type T4 phage	*Raw beef*	A total of 25 g of meat sample was inoculated with diluted culture bacteria overnight and allowed to attach for 10 min at room temperature. Phages immobilized on cellulose membranes were used to cover the contaminated surface of the meat.	The use of immobilized phages resulted in the reduction of 1 log unit after 6 and 9 days, and below detection limit on days 12 and 15 at 4 °C.	[121]
Cocktail composed of phages DT1 to DT6	Milk and meat	Sterile, commercial, reconstituted milk supplemented with CaCl_2_ was inoculated with each bacterial strain (one per batch). Each batch was divided into 2: one treated with phage cocktail and other to be the control. Meat pieces (1 cm, 0.4 cm thick) were spotted with bacterial strains and allowed to attach for 10 min at room temperature. Then, phage cocktail was added to each meat piece. Controls were prepared by adding TMG buffer.	Phage cocktail could reduce the amount of *E. coli* strains tested in milk with reduction values reaching up to 3.4 log_10_ CFU/mL at 24 °C and 3.6 log_10_ CFU/mL at 37 °C after 24 h. In meat, cell inactivation was achieved at 24 °C (2.6–4.0 log_10_ CFU/mL) and 37 °C (3.0–3.8 log_10_ CFU/mL) as well. A higher inactivation value for O157:H7 STEC (1.55 ± 0.35 log_10_ CFU/mL) was observed at 4 °C after 6 days.	[122]
Ecoshield	Lettuce	Overnight cultures were applied to fresh cut lettuce. Phages were applied by immersion or spraying in combination with sodium hypochlorite solution.	Spraying of phages on lettuce surface reduced the *E. coli* O157:H7 populations (2.22 log CFU/cm^2^) compared with control treatments (4.10 log CFU/cm^2^), while immersion of lettuce in suspensions containing high concentrations of EcoShield™ (9.8 log PFU/mL) resulted in the deposition of high concentrations (7.8 log PFU/cm^2^) of bacteriophages on the surface of fresh cut lettuce, thereby contributing to the efficacy of the lytic phages on lettuce.	[123]
FAHEc1	UHT milk; ready-to-eat meat; raw beef	Phage FAHEc1 was treated with UV light before being used on food products (even losing viability, phages are capable of lysing bacterial cells). UHT milk was inoculated with *E. coli* O157:H7 and phages. The raw beef inoculation was at 37 °C and was used to simulate the phage application right before slathering in carcasses.	In comparison to UHT milk and raw beef, there was a greater reduction produced by viable phages with a reduction value of 4.5 log_10_ CFU piece^−1^ after 24 h incubation. In milk, UVP produced a 2–2.5 log_10_ CFU reduction, while in raw beef, UVP produced a reduction of 1.75–2.5 log_10_ CFU.	[124]
Phages T5, T1, T4, and O1	Beef	Beef portions were placed in a sterile petri plate and *E. coli* O157 was added. After 10 min (for samples to dry) phages (individually or in cocktail) were added and compared at different time and temperature. PBS was used as control.	Effect of temperature and time showed a reduction in *E. coli* O157 numbers by 3.2 log_10_ CFU/cm^2^ compared to phage-free controls at 4 °C after 144 h, the same reduction was observed at 22 °C and 37 °C but after 6 and 3 h, respectively.	[125]
phiEco1, phiEco2, phiEco3, phiEco5, phiEco6 and phiS1	Oyster	Bacteria grown overnight were added to the oysters and allowed to attach for 1 h at 37 °C. Phage suspension was added, and the oyster meat was then incubated at 3 °C for 2 days followed by an incubation at 37 °C for 2 h.	High concentration of phages could reduce all bacteria strains. The reduction was observed when bacteria were present in single or combined manners. Phages were able to reduce *E. coli* (ATCC BAA-196) concentration from 8.5 ± 3.5 × 10^7^ CFU/g oyster meat to 2.0 ± 1.5 × 10^6^ CFU/g oyster meat even after 50 h of incubation.	[126]
*E. coli* O157:H7 phage isolated by Cui et al. (2018)	Lettuce Cucumber and carrot	The different vegetables were immersed in *E. coli* suspensions for 30 min and then placed at 37 °C for 24 h. The vegetables were then sequentially treated with Cold Nitrogen Plasma (CNP) and phages.	The sequential treatment led to a reduction in cell viability to 1.21 log_10_ CFU/g on the third day and no viable bacteria was detected on the ninth day. The results were independent of temperature (4, 12, or 25 °C).	[127]
*E coli* O157:H7 phage	Spinach; spinach harvester blade	Spinach extract was prepared using 25 g fresh spinach leaves. The Blades were inoculated with the extract or 10% TSB (control). Two Different conditions were used at a static temperature (22 °C) or dynamic temperature (30 °C 12 h, 20 °C 12 h), to stimulate day and night temperatures in California. Blades inoculated with *E. coli* were treated with phages.	phages could successfully eliminate *E. coli* O157:H7 population on the blades after 2 h exposure with a 4.5 log CFU reduction. After 24 and 48 h incubation at 30 °C, growth was significantly higher (6.09 and 6.37 log CFU/mL) than when incubated at 22 °C (4.84 and 5.68 log_10_ CFU/mL) respectively.	[128]
Phages phiJLA23, phiKP26, phiC119, and phiE142	Tomatoes	A phage cocktail containing 10^9^ PFU/mL of each phage. Microencapsulated phages were also prepared containing a polymer mixture consisting of 10% solids (modified starch and maltodextrin), 60% of SM buffer and 30% of phage cocktail. Tomatoes were divided into 3 groups: 1st group was inoculated with *E. coli* O157:H7; 2nd group was inoculated with microencapsulated cocktail phage and sprayed with the bacteria host, and the 3rd was not inoculated (control).	After 120 h at 4 °C, microencapsulated phages could significantly reduce *E. coli* O157:H7 concentrations from 5.2 Log_10_ CFU/tomatoes at 0 h to 2.3 Log_10_ CFU/tomatoes. Results obtained showed that microencapsulated phages are more stable under stress factors than the free phages.	[129]

### 3.4. Safety Attributes of Bacteriophages for Biocontrol of E. coli O157:H7

Recent studies have revealed a global resurgence of interest in phages as potential therapeutic agents for clinically relevant bacterial infections, especially those caused by multi-drug-resistant strains [130,131]. This renewed interest in phages is attributed to some advantages they have over conventional antibiotics.

Phages have the ability to selectively target and kill specific bacterial groups while preserving commensal microbiomes, which results in a reduced number of bacteria cells being subjected to selection pressure [132]. This specificity reduces the risk of phage-based biocontrol causing damage to beneficial bacteria or non-target organisms in the environment, thereby enhancing its safety profile. Aside from the fact that phages can replicate at infection sites and penetrate biofilms [133], extensive research has demonstrated that phages are generally non-toxic and harmless for human consumption [134] unlike antibiotics, which can lead to the development of antibiotic-resistant bacteria if mis-used.

Furthermore, phages are self-limiting; their population tends to decrease as the amount of their host bacteria decreases [135]. They ultimately degrade in the environment, thereby reducing concerns regarding persistence and accumulation over time. This property helps prevent the overgrowth of phages when they are used as a biocontrol, thereby minimizing their effect on the bacterial community. Finally, bacteriophages can be found in a variety of environments, including water sources, sediments, and the digestive tracts of animals [136], hence isolating them is not a difficult task in terms of their availability.

### 3.5. Bacteriophage Therapy and Phage-Based Products for the Control of E. coli O157:H7

Phage therapy is a method that uses phages to effectively treat bacterial infections in medicine; this is made possible by the abundance of phages in nature, their simplicity in isolation, and the efficiency with which they kill bacteria, especially when used in controlled laboratory research. The emergence of AMR has made it possible to investigate phages as an antibiotic alternative. *E. coli* phages have been created for oral administration and utilised in randomised trials [137,138]. Currently, researchers are attempting to employ phages in a dried powder form that is considerably more stable and easier to store, transport, and administer. Despite the research on phage therapy, there are currently no phage therapy products licenced for human use in the EU or the US. To manage *E. coli* O157:H7 in the food sector, phage-based products have been developed to be used as biocontrol of bacterial diseases and have been authorised by the FDA as “generally considered safe.” Examples of phage-based anti-*E. coli* O157:H7 products include the following; EcoShield^TM^, originally designed for application in “red meat parts” [139], and Secure Shield E1, a product manufactured by FINK TEC GmbH, a German company, that is specifically designed for application on the external areas of beef carcasses [140]. Every product has obtained approval from the Food and Drug Administration (FDA). Several food products have recently received approval for the utilization of phage mixes from OmniLytics (Sandy, UT), Micreos’ PhageGuard E., and Intralytix’s EcoShield PXTM to mitigate the risk of infection caused by *E. coli* O157:H7 and other pathogenic *E. coli* strains [141] (Table 3).

The FDA has granted approval for the phage products to be classified as generally recognized as safe (GRAS) food additives. The GRAS categorization mentioned above is derived from the regulatory framework established by the Federal Food, Drug, and Cosmetic Act (FD&C Act) and the regulations outlined in Title 21 of the Code of Federal Regulations (21 CFR). In order to introduce novel chemicals into the human food supply, particularly those intended for use as food additives, it is necessary to adhere to the regulatory guidelines outlined in 21 CFR part 170, subpart E [142]. This procedure has been used for the purpose of certifying bacteriophages, predominantly in the United States.

**Figure 3:**
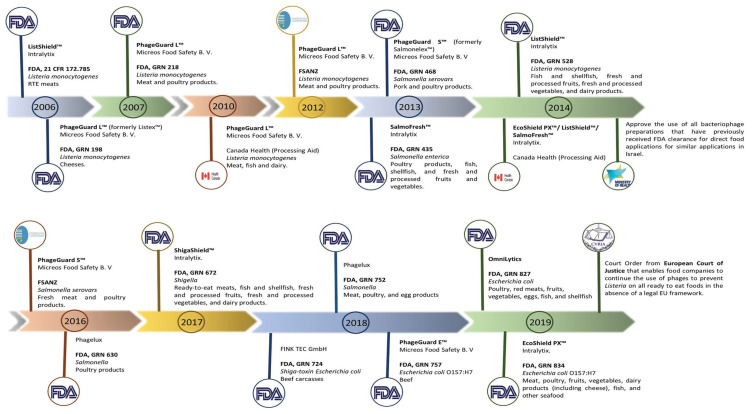
Phage-based products that have already received approval around the world. The manufacturer, the target microorganisms, and the food matrix where the product is intended to be applied are all listed in the product description. The FDA, FSANZ, and the companies’ news websites were used to create the timeline. Adapted with permission from: Pinto et al. [80], 2023, Taylor & Francis Ltd., http://www.tandfonline.com, 1 September 2023.

Other nations have also granted approval for the use of bacteriophages to ensure the safety of food products. In 2014, the Israeli Ministry of Health’s National Food Service authorized the utilization of bacteriophages, which had previously received approval from the FDA, for similar applications. This decision was made in accordance with the regulations outlined in the document titled “Guidelines: use of bacteriophages (bacteria-killing viruses) in food” [143]. In Canada, the authorization for the use of phage-based products as food processing aids has been granted. These products include PhageGuard LTM (previously known as Listex^TM^), ListShield^TM^, SalmoFresh^TM^, and EcoShield^TM^, among others [143] (Figure 3).

## 4. Challenges Faced in the Application of *E. coli* O157:H7 Phages

There are a number of difficulties and factors to take into account when using phages as a biocontrol for *E. coli* O157:H7 either on food products or as a medication in humans and animals. Phages are very specific to individual bacterial strains [144]. Since *E. coli* O157:H7 strains can vary genetically and develop resistance to phages over time, it can be difficult to find phages that effectively target these strains. As a result, a combination of different phages in the form of a cocktail may be necessary for effective biocontrol of this pathogen. *E. coli* O157:H7 has a low infectious dosage (10 to 100 CFU/mL) and can survive in low-pH conditions, such as the acidic environment of the stomach and acidic food, which is one of its stress resistance mechanisms [145]. It can be therefore difficult to deliver phages to the site of infection because they may need to endure stomach acid and other digestive processes in order to reach the intestinal *E. coli* O157:H7 in the gut. For phages to be considered effective at inhibiting *E. coli* O157:H7, they must possess acid resistance in order to survive in the acidic environment of the stomach. Furthermore, phage products can be expensive to create, produce, and distribute due to their sensitivity to environmental factors like pH and temperature [146]; their quality and effectiveness can be compromised if they are transported and stored in unsuitable environmental conditions. The issue of storage and transportation has made it difficult for patients situated in low-resource environments to have access to them. Additionally, research has shown that phage therapy may be more effective when combined with other treatments, such as antibiotics [147,148]. Since its usage in humans could raise ethical concerns, it is crucial to carefully consider the best sequence and combination of therapies that will not cause further complications in infected patients. Inadequate clinical data also pose a challenge in the application of *E. coli* O157:H7 phages; while there have been some case reports and small clinical studies on phage therapy [149], more large-scale, randomised controlled trials are required to establish the safety and efficacy of phages as a biocontrol agent in treating *E. coli* O157:H7 infections.

### Phage Resistance by E. coli O157:H7

The evolution of phage resistance in *E. coli* O157:H7 is a continuous and natural process. As a result of repeated use of phages against *E. coli* O157:H7, in vitro experiments have shown that *E. coli* O157:H7 can develop phage resistance [150]. To avoid phage infection, they employ a variety of processes that involve the following techniques.

Phage inability to attach: *E. coli* O157:H7 may alter the architecture of its cell surfaces to restrict phages from attaching to its surface [151]. To attach to particular receptors on the bacterial cell wall, phages often use receptor-binding proteins [152]. Phage attachment may be prevented by changes to these receptors. *E. coli* O157:H7 can change its surface characteristics, such as the lipopolysaccharide (LPS) layer, thereby making it challenging for phages to adhere to the bacterial cell [153].

CRISPR-Cas Systems: Some *E. coli* O157:H7 strains have Clustered Regularly Interspaced Short Palindromic Repeats (CRISPR) and CRISPR-associated (CRISPR-Cas) systems, which are a form of adaptive immunity against phages. CRISPR-Cas systems play a significant role in limiting phage infection and proliferation as an important bacteriophage resistance mechanism [154]. By using these systems, bacteria can collect and preserve genetic material from earlier phage contacts and use this information to locate and eliminate the phage DNA when there is a contact with the same phage.

Restriction–Modification (R-M) Systems: *E. coli* O157:H7 and other organisms may have R-M systems. These systems are made up of enzymes that protect bacterial cells by breaking down foreign DNA (including phage DNA) that enters the cell. They recognize and cleave phage DNA sequences on the recognition site [155], thereby inhibiting phage genetic material from multiplying.

Abortive Infection: The infected *E. coli* strain employs abortive infection mechanisms, resulting in self-destruction prior to the completion of the phage’s replication cycle [156]. This behaviour effectively inhibits the phage epidemic from spreading to neighbouring cells, thus ensuring the safety of the bacterial colony.

Understanding the mechanisms of phage resistance is critical in phage therapy. The mechanisms described above are strategies adopted by numerous bacterial populations to protect themselves from phage predation, and they can differ amongst strains of *E. coli* O157:H7. Due to the pressure exerted by phages, the pathogen can develop resistance to phages over time. As a result, phage cocktails can be used as a method to target multiple mechanisms or in combination with other treatment options to overcome bacterial resistance.

## 5. Determining the Safety of *E. coli* O157:H7 Specific Bacteriophages as a Biocontrol Agent

To maximise the potential of phage treatment of *E. coli* O157:H7 infections, phage candidates must be safe and capable of serving as biocontrol agents without having negative effects on people or the environment.

Firstly, phage’s specificity for *E. coli* O157:H7 must be ensured by isolating and properly characterising candidate phage; afterwards, phages should be screened for the existence of genes linked to virulence and antibiotic resistance within the phage’s genome simply because genes that encode toxins and allergens must not be present in phages intended for biocontrol as they can be detrimental to humans and animals [108]. New generation sequencing techniques like the Whole Genome Sequencing (WGS) can be used to analyse the phage genome and determine the presence of these genes.

WGS is a method of typing that relies on sequencing of the entire genome of an isolate, allowing for the identification of variations at the level of individual nucleotides [157]. Based on the findings of the study conducted by Lee et al. [158], WGS was used to determine the absence of virulence factors, toxins, antibiotic resistance, and potential allergen-coding genes in the genome of the isolated phage KFS-EC. The phage which was obtained from wastewater samples from a slaughterhouse in Korea exhibits specificity exclusively towards *E. coli* O157:H7 and was considered to be suitable as a biocontrol agent. In addition, as described by Liao et al. [159], WGS can be used to determine the presence of lysogenic elements that may potentially enable the dissemination of undesirable genes among bacterial communities. Having confirmed the lack of such elements, the phages can then be considered to be safe for use as a biocontrol. It is vital to highlight that WGS supports the “One Health” concept, which considers the interrelated nature of human, animal, and environmental health. This is demonstrated by the impact WGS has on public health responses, as its usage can quickly pinpoint the safety of phage candidates and support the implementation of efficient control measures to stop the spread of antimicrobial resistant microbes [160].

Having considered the genomic screening of phages, conducting animal studies to assess the safety of the phages in the target host organisms (e.g., livestock) is essential before a large scale production. Studies on the effectiveness of phages in treating experimentally infected animals [161,162] have demonstrated that using mice, chickens, or sheep in biocontrol trials will allow for the monitoring of any negative effects on animal health [163]. Clinical trial studies can evaluate the safety of phage therapy in people, including possible allergic reactions or other adverse effects. They can also determine the right concentration and dosage of phages required to control *E. coli* O157:H7 in people.

Due to the sensitive nature and ethical issues associated with phage application, stakeholders like farmers, food processors, and healthcare professionals should obtain training on how to handle phages safely and responsibly in the real-world settings. Furthermore, while ensuring phage purity and quality during preparation, there is a possibility of generated residues and contaminants causing environmental hazards, which could pose a health risk. It is crucial to monitor the potential environmental impact of using phages, such as phage persistence in the environment and potential ecological disruptions.

In the event that genetic modifications in the host bacterium impart phage resistance, such phages are not suitable for use as a biocontrol. Therefore, phage safety concerns require constant monitoring and surveillance. For example, it is important to use appropriate testing methods (such as microbiological assays) to quantify pathogen levels before and after phage application, monitor the development of phage resistance, regularly evaluate the effectiveness of phage treatments in controlling target pathogens like *E. coli* O157:H7, and monitor the safety and efficacy of phage products once they are on the market.

## 6. Conclusions and Future Perspectives

In the current era of antimicrobial resistance and the pursuit of alternative methods to combat *E. coli* O157:H7 infections, there has been a renewed interest in phage research. This is evident in the accumulated evidence suggesting that phage application is an effective method of biocontrol of foodborne bacterial pathogens on fresh produce and other foods. Phages has been used as a natural antimicrobial method to reduce *E. coli* O157:H7 from the food supply. Phage-based medications have been approved by the Food and Drug Administration (FDA) in certain countries, such as the United States, Canada, and Israel for the treatment of E. coli strains, including the use of various phage cocktails. Numerous studies have also produced novel phages and showed their effectiveness in inhibiting the growth of *E. coli* O157:H7 in food products without changing the organoleptic properties. They have also been proven to reduce *E. coli* O157:H7 in vivo through the use of mice. Ongoing research involves the construction of phage libraries, thereby enabling the use of phage cocktails to inhibit phage mutant strains, increase host range, and increase infectivity. Nevertheless, the European Union remains concerned about the utilization of these products due to the limited availability of safety data and comprehensive research on the potential consequences of phage discharge into the environment. One of the most important aspects of creating One Health approaches to lowering health risks for people, agricultural systems, and natural ecosystems is the effective use of “safe” phages as a biocontrol agent in combating pathogens like *E. coli* O157:H7 and resolving the issue of antibiotic resistance in preharvest livestock environments. In order to advance the development of novel antibacterial strategies, there is a need to conduct further investigation into the phage properties that make them safe and suitable as a biocontrol and the underlying mechanism through which phages can effectively inhibit the expression of host genes. The utilization of advanced technologies like WGS to carefully scrutinize and characterize phage genomes presents a solution to this concern. This, in turn, will improve both patient care and infection control strategies, ultimately leading to a decrease in the incidence of severe *E. coli* O157:H7 infections. This review highlights the renewed hope provided by modern technologies in combating *E. coli* O157:H7 infections, reducing the persistence and spread of antimicrobial resistance, and an enhanced potential in improving livestock production. These further provide an indication of increased food security and safety, and thus a significant contribution towards achieving the goals of the highly integrated “One health” approach.

## Figures and Tables

**Table 3:** Approved phage-based products for *E. coli* O157:H7.

	Bacteriophage	Applications	Regulatory Approval
1	PhageguardE	Beef	USDA/FDA/GRN 757
2	EcoShield PX Intralytic, Inc., Columbia, MA, USA	Meat, poultry, food, vegetables	FDA (2011)/GRN 834
3	Omnilytics	Poultry, red meat	FDA/GRN 827
4	SecureShield E1, FinkTec GmbH, Hamm, Germany	Beef carcasses	FDA/GRN 724

## Data Availability

Not applicable.
